# Jasmonic Acid as a Mediator in Plant Response to Necrotrophic Fungi

**DOI:** 10.3390/cells12071027

**Published:** 2023-03-27

**Authors:** Violetta Katarzyna Macioszek, Tomasz Jęcz, Iwona Ciereszko, Andrzej Kiejstut Kononowicz

**Affiliations:** 1 Laboratory of Plant Physiology, Department of Biology and Plant Ecology, Faculty of Biology, University of Bialystok, 15-245 Bialystok, Poland; 2 Faculty of Biology and Environmental Protection, University of Lodz, 90-237 Lodz, Poland; 3 Department of Plant Ecophysiology, Faculty of Biology and Environmental Protection, University of Lodz, 90-237 Lodz, Poland

**Keywords:** circadian clock, COI1, defense responses, jasmonates, necrotrophic fungi, signaling, resistance

## Abstract

Jasmonic acid (JA) and its derivatives, all named jasmonates, are the simplest phytohormones which regulate multifarious plant physiological processes including development, growth and defense responses to various abiotic and biotic stress factors. Moreover, jasmonate plays an important mediator’s role during plant interactions with necrotrophic oomycetes and fungi. Over the last 20 years of research on physiology and genetics of plant JA-dependent responses to pathogens and herbivorous insects, beginning from the discovery of the JA co-receptor CORONATINE INSENSITIVE1 (COI1), research has speeded up in gathering new knowledge on the complexity of plant innate immunity signaling. It has been observed that biosynthesis and accumulation of jasmonates are induced specifically in plants resistant to necrotrophic fungi (and also hemibiotrophs) such as mostly investigated model ones, i.e., *Botrytis cinerea*, *Alternaria brassicicola* or *Sclerotinia sclerotiorum*. However, it has to be emphasized that the activation of JA-dependent responses takes place also during susceptible interactions of plants with necrotrophic fungi. Nevertheless, many steps of JA function and signaling in plant resistance and susceptibility to necrotrophs still remain obscure. The purpose of this review is to highlight and summarize the main findings on selected steps of JA biosynthesis, perception and regulation in the context of plant defense responses to necrotrophic fungal pathogens.

## 1. Introduction

Jasmonates (JAs) are one of the structurally simplest plant hormones. The term ‘jasmonates’ describes the group of oxylipin phytohormones, derivatives of jasmonic acid (JA), that come into existence in cytosol, such as methyl ester of JA (MeJA), *cis*-jasmone, jasmonic acid glucoside (JA-Glc), 12-hydroxyjasmonic acid (tuberonic acid, 12-OH-JA) or JA-isoleucine conjugate (JA-Ile), that regulate diverse developmental and physiological processes [1,2]. JA plays multifarious roles in plant physiological processes, i.e., growth and development [3], circadian rhythm of metabolism [4], senescence and cold acclimation [5], as well as the response to abiotic and biotic stresses [6,7]. The special function, however, of jasmonic acid is performing as a signal mediator in defense against herbivorous insects [8] and necrotrophic pathogens [9]. During plant defense response, JA not only induces the expression of pathogenesis-related (PR) genes [10] but also regulates the secondary metabolism promoting synthesis of flavonoids, glucosinolates, terpenoids and phytoalexins [11,12], as well as lignin deposition that enhances the mechanical structure of cell walls [13,14]. Jasmonates levels vary depending on plant species and environmental conditions; thus, their concentration in response to stress is an individual quality of a plant [15].

The hormonal character of jasmonates, although hitherto widely accepted, was ultimately confirmed by the discovery of the JA-specific receptor complex. CORONATINE INSENSITIVE1 (COI1) protein, first described by Xie et al. [16], was proven to bind directly to JA-Ile and serve as a jasmonate receptor [17]. The bioactive form of JA-isoleucine conjugate is (+)-7-iso-JA-L-Ile, whereas its (-)-JA-L-Ile epimer was shown to be an inactive, although more stable, form. The pH changes promote conversion of (+)-7-iso-JA-L-Ile to the inactive (-)-JA-L-Ile form, thus providing a simple mechanism that can rapidly and reversibly regulate hormone activity through epimerization [18]. The perception of JA-Ile conjugate is crucial for interaction of the COI1 and Jasmonate-ZIM (Zinc-finger Inflorescence Meristem) domain (JAZ) repressor protein. It was proven, that in Arabidopsis and tomato only that this form of jasmonate, unlike the other JA derivatives such as methyl jasmonate (MeJA) or JA precursor 12-oxo-phytodienoic acid (OPDA), promotes binding JAZ1 by COI1 [19,20]. Forming the COI1-JAZ1 complex does not involve any JA-Ile-induced enzymatic modifications, as JA-Ile promotes the direct physical interactions between these two proteins [21].

Contrary to biotrophic pathogens that feed on living host tissues, necrotrophic fungi obtain nutrients by killing plant cells and feeding on dying or dead host tissues. Necrotrophic fungal pathogens attack either a broad spectrum of host plant species or a narrow host range, or even, rather rarely, like many biotrophic fungi, a single host plant species [22,23]. Necrotrophic fungi cause substantial crop yield loss during all steps of crop agriculture from seed, through seedlings and young plant stages, to mature, ready to harvest, plants and also postharvest during storage. Moreover, they generate more devastating economic impact on food production world-wide than biotrophic fungi [24,25]. Extensively studied model necrotrophic fungi such as generalists *Botrytis cinerea* [26] and *Sclerotinia sclerotiorum* [27] or a specialist that infects plants of *Brassicaceae* family—*Alternaria brassicicola* [28]—induce JA pathway in resistant and to a lesser extent also in susceptible host plants. Infection of plant host cells by a necrotrophic fungus is accomplished mostly either by a repertoire of fungal cell wall degrading enzymes (CWDE) and plethora of toxins or by a more intricate mechanism containing secreted effector proteins and plant receptors, although this last possibility is only currently being broadly discussed and supported by genomic studies in regard to necrotrophic fungi [29]. Upon perception of necrotrophic fungi by host cellular receptors, signal transduction through secondary messengers (e.g., reactive oxygen species, ROS) triggers plant resistance responses leading, among other events, to JA biosynthesis and activation of a JA-dependent signaling cascade including a set of transcription factors (TFs) and following over-expression of defense-related JA marker genes such as, e.g., plant defensin (e.g., *PDF1.2*) and/or thionin (e.g., *THI 2.1*) [30,31,32]. The complexity of not yet fully explored JA-dependent defense responses of plants to necrotrophic fungi and the possibility of using their many features in contemporary agricultural technologies as an alternative to for example fungicides is one of the most interesting areas in modern plant science.

In this review, we aim to highlight selected steps of JA biosynthesis, perception and regulation in regard to plant response specifically to necrotrophic fungal pathogens. Moreover, JA regulation by circadian clock and photoperiod, its trade-off between growth and defense, as well as JA and ethylene (ET) cooperation during infection have been described.

## 2. Biosynthesis of Jasmonates Induced in Response to Necrotrophs

Biosynthesis of different jasmonate classes has been comprehensively described in reviews by Lyons et al. [33], Wasternack and House [34] and Yan et al. [35]. Here, the JA biosynthesis steps will be considered exclusively in the context of plant responses to necrotrophic fungus infections.

**Figure 1:**
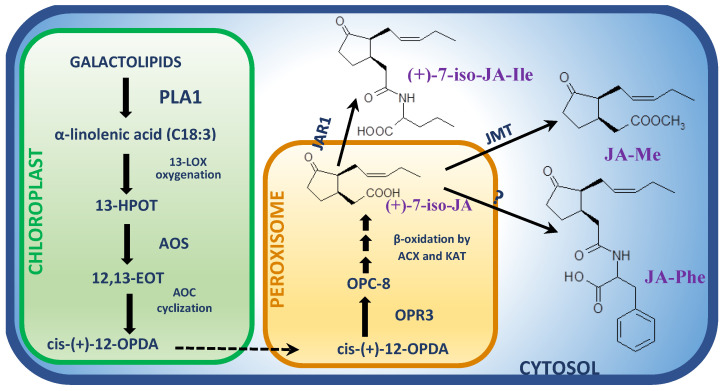
Biosynthesis pathway of jasmonates in Arabidopsis active during necrotrophic interactions [1,2,3,9,30,32,33,34,35]. Detailed description in the text. Abbreviations: ACX, acyl-CoA oxidase; AOC, allene oxide cyclase; AOS, allene oxide synthase; 12,13-EOT, 12,13(S)-epoxy-9(Z),11,15(Z)-octadecatrienoic acid; (+)-7-iso-JA, (+)-7-iso-jasmonic acid; (+)-7-iso-JA-Ile, (+)-7-iso-jasmonoyl-L-isoleucine; JMT, JA carboxyl methyl transferase; JAR1, Jasmonate-Resistant synthase; JA-Me, methyl ester of JA; JA-Phe, jasmonoyl-phenylalanine; 13-HPOT, (13S)-hydroperoxy-octadecatrienoic acid; KAT, l-3-ketoacyl-CoA thiolase; 13-LOX, 13-lipoxygenase; OPC-8, 3-oxo-2-(2′-[Z]-pentenyl)cyclopentane-1-octanoic acid; cis-(+)-12-OPDA, cis-(+)-12-oxo-phytodienoic acid; OPR3, 12-oxo-phytodienoic acid reductase; PLA1, phospholipase A1.

The preferential precursor of JA biosynthesis is tri-unsaturated fatty acid, α-linolenic acid (C18:3; α-LeA) (Figure 1), and JA is rapidly synthesized from it upon necrotrophic fungi attack in many pathosystems [1,34]. The elevated content of both α-LeA and JA in *Fusarium graminearum*-infected wheat bearing the Fhb1 (*Fusarium* head blight) quantitative resistance locus has been observed [36]. Increased levels of α-LeA and JA-Ile have also been noticed as characteristic features in both resistant and susceptible soybean cultivars infected with *S. sclerotiorum*, although α-LeA level was relatively higher in infected resistant cultivar. In the case of JA-Ile, its level increased in infected cultivars with different cultivar- and time-dependent dynamics. In resistant soybean cultivar, elevated JA-Ile concentration at an early stage of infection (at 6 hpi, hours post-inoculation) has been observed, whereas in susceptible soybean cultivar, JA-Ile level increased only later at 48 hpi [37]. Interestingly, in the above-mentioned study, *F. graminearum*-infected resistant Fhb1 wheat cultivar showed only a slight increase of endogenous JA content at an early stage of infection, which returned to the control level at 12 hpi, but in susceptible wheat cultivar, the infection caused a decrease of JA content compared to the control from 24 hpi [38].

Plant phospholipases (PLs) are grouped into three main classes with different families and subfamilies according to their DNA sequences, i.e., phospholipases A (PLAs), C (PLCs) and D (PLDs) [39]. Phospholipases A hydrolyze glycerophospholipids to release lysophospholipids and free fatty acids at the *sn*-1 (PLA1) and/or *sn*-2 position (PLA2) of glycerolipids [40]. Phospholipases C and D act on polar heads of phospholipids; PLCs hydrolyze the phosphodiester bond of the glycerol side to generate diacylglycerol and phosphorylated head groups; and PLDs hydrolyze the phosphodiester bond at the head group side to produce phosphatidic acid and free head groups [39,41]. Thus, it is widely accepted that enzymes of phospholipase A1 family are responsible for α-linolenic acid release from galactolipids of chloroplast membrane (Figure 1) [1,34,39]. Nonetheless, it has been reported that the induction of basal JA accumulation in Arabidopsis phospholipase A1-knockout lines was not impeded upon *B. cinerea* infection [42]. However, increased susceptibility to *B. cinerea* and significantly lower JA content (and also lower phosphatidic acid production) compared to wild type plants were revealed in phospholipase Dβ1-deficient Arabidopsis mutants, suggesting the significant role of phospholipase D1 in host resistance to the necrotrophic fungus and its positive role in the pathogen-induced JA biosynthesis [41,43]. It seems that PLC does not affect JA production during plant-necrotrophic fungi interactions [41], but a mechanism in which PLD affects JA biosynthesis remains to be determined.

The initial step in JA biosynthesis is oxygenation of α-linolenic acid in the C-13 position by lipoxygenase (LOX) (Figure 1) [34]. Tomato mutants impaired in TmLOXD (wound-induced 13-lipooxygenase) function were unable to produce JA. Moreover, the significantly increased accumulation of JA as well as enhanced resistance to *B. cinerea* in tomato plants overexpressing *LOXD* gene was observed [44]. The fatty acid hyperoxide resulting from α-LeA oxygenation is subsequently dehydrated by allene oxide synthase (AOS) to unstable allene oxide. In the presence of allene oxide cyclase (AOC), allene oxide is transformed into 12-oxo-phytodienoic acid (OPDA) enantiomer, 9S,13S/cis-(+)-OPDA, and it is the last step of JA biosynthesis that takes place in chloroplasts (Figure 1) [2]. The role of AOC in JA-dependent response to necrotrophic infection was confirmed in the *Oryza sativa*–*Magnaporthe oryzae* pathosystem, in which the rice mutants impaired in AOC production showed reduced production of JA and increased susceptibility to the pathogen [45]. In peroxisomes, cis (+)-OPDA is further converted into (+)-7-iso-JA by 12-oxo-phytodienoic acid reductase (OPR) and three β-oxidation steps involving acyl-CoA oxidase (ACX) and l-3-ketoacyl-CoA thiolase (KAT) (Figure 1) [1,2]. Tomato plants with a silenced *OPR3* gene displayed a significant increase in susceptibility to *B. cinerea* accompanied by the dramatically decreased production of both OPDA and JA-Ile [46]. Consequently, in double *opr7/opr8* maize (*Zea mays*) mutants, the reduced biosynthesis of JA as well as a diminished resistance to oomycete *Pythium aristoporum,* was observed [47].

In the next step of JA biosynthesis in cytosol, (+)-7-iso-JA may be subsequently conjugated with an amino acid by JAR1 (JASMONATE RESISTANT1) synthase, which is able to bind amino acids exclusively to jasmonic acid molecule (Figure 1) [48]. Different members of JAR family may synthetize rather rarely the JA conjugates with different amino acids such as valine (Val), leucine (Leu) and phenylalanine (Phe) [30]; however, the most biologically substantial conjugate JA-Ile is provided by JAR1 [49]. The Arabidopsis *jar1* mutant showed increased susceptibility to both the *S. sclerotiorum* strain deprived of *Sclerotinia sclerotiorum* integrin-like (SSITL) protein suppressing host resistance as well as to the wild type *B. cinerea* isolate [50]. Accordingly, in rice plants challenged with *Magnaporthe grisea* infection, a gradual increase in expression of *OsJAR1*, but not the *OsJAR2* gene, was observed from 48 to 72 hpi. Simultaneously, the elevated *OsJAR1* expression was accompanied by induction of endogenous JA-Ile, but not JA-Phe levels, within the same time period [51]. In agreement with the above findings, the content of (+)-7-iso-JA-Ile was found to be significantly elevated in wheat Fhb1 plants inoculated with *F. graminearum* in comparison to mock-inoculated plants [36], providing yet more evidence for the JA-Ile as a crucial jasmonate in defense against necrotrophic fungi. Metabolite profiling studies of Arabidopsis plants infected with *B. cinerea* showed the maximum peak of JA-Ile accumulation at 3 days post-inoculation (dpi) [52]. The intensity and duration of JA responses are controlled to a large degree by the precise balance between biosynthesis and catabolism of JA-Ile. It was demonstrated that *CYP94B3*, *CYP94C1* and *CYP94B1* genes, the members of Cyt P450 family, play a key role in JA-Ile catabolic inactivation [53,54,55,56]. These genes encode JA-Ile 12-hydroxylase, which is an enzyme catalyzing the conversion of JA-Ile to biologically inactive hydroxylated forms. The disease symptoms in *B. cinerea*-infected Arabidopsis lines overexpressing *CYP94B3* and *CYP94C1* genes (B3-OE and C1-OE, respectively) were much stronger in comparison to wild type plants. Moreover, the expression levels of JA defense cascade marker genes, *PDF1.2* and *PR4*, were strongly impaired in infected OE lines. These findings clearly indicate that CYP94B3 and CYP94C1 are integral components of the fungus-induced metabolic pathway controlling the abundance of JA-Ile [52]. In general, JA and its precursors contents increase in plant cultivar resistant to necrotrophic fungi more than in susceptible ones.

In the context of defense response against necrotrophic fungal infection, the concurrent/independent operation of another jasmonate forms alternative to JA-Ile conjugate should be considered. Analogous yet variant phenomenon revealed the significant accumulation of JA-Phe conjugate and its cyp94-oxidized forms in Arabidopsis plants infected with *B. cinerea*, suggesting that precisely controlled levels of JA-Phe may also be involved in responses to necrotrophic pathogens [57]. In maize, infection by *Cochliobolus heterostrophus* resulted in the local production of 9-lipoxygenase (LOX)-derived 10-oxo-11-phytoenoic acid (10-OPEA), 10-OPDA and a series of related 12- and 14-carbon cyclopente(a)nones, which apart from displaying direct phytoalexin activity, mediate defense gene expression [58]. Similarly, in tomato plants infected with *B. cinerea*, OPDA played a major role in defense response not only as a precursor of JA but also as an autonomous mediator [46].

The role of methyl ester of jasmonic acid (MeJA) as a mediator in defense against necrotrophs was also suggested [59]. Only a few studies have provided, however indirectly, further evidence supporting this theory. Fungal elicitor alamethicin isolated from *Trichoderma viridae* was revealed to cause significant induction of gene encoding JA carboxyl methyl transferase (JMT), a key enzyme catalyzing the conversion of JA to MeJA, in poplar (*Populus trichocarpa*) leaves within 2 h after treatment [60]. Consistently, the transcriptional activation of JMT was observed in *Brassica juncea*-*Alternaria brassicicola* pathosystem at 2 dpi [61]. However, it has to be emphasized that an exogenous application of MeJA to plants before or simultaneously with a necrotrophic fungus induce in many pathosystems a sufficient defense response to restrict a necrotroph development and limit lesions spreading [62,63,64,65,66].

Regardless of their experimentally confirmed function in defense response to necrotrophic fungi, the molecular mode of action, possible hormonal character and perception mechanism of jasmonate forms alternative to JA-Ile remain obscure.

## 3. JA Biosynthesis Genes Induced in Response to Necrotrophic Fungi Infection

The need for accumulation of JA levels effective for signal transduction in response to pathogen infection compel host plants into reprogramming the transcriptional activity of JA-biosynthesis genes. Accordingly, numerous transcriptomic surveys confirmed that genes encoding enzymes involved in JA biosynthesis are induced upon necrotrophic fungi infection, suggesting the direct and pathogen-responsive transcriptomic regulation of JA abundance *in planta*. Below, the expression profiles of selected JA-biosynthesis genes are revised in this context.

### 3.1. Phospholipase (PL) Genes

As mentioned above, the primary role of A1 family phospholipases is releasing α-linolenic acid for further JA biosynthesis; although possible, this seems to be uncertain in the case of response to necrotrophic fungi infection. However, deep transcriptome sequencing experiments revealed the significant up-regulation of *PLA1* genes upon pathogen attack, the involvement of yet another *PLs* gene has to be considered in at least JA biosynthesis regulation. In Arabidopsis plants infected with *B. cinerea,* the induction of *A1* as well as *Dγ1* phospholipase genes (observed at 18 hpi), was preceded by the up-regulation of *PLA2* gene (12 hpi), whereby the elevated levels of all these genes transcripts were detectable also at 24 hpi [67]. In earlier research on the same pathosystem, no significant up-regulation of *A1* family phospholipase genes has been observed. However, induction of *A2α, Dγ1* and *Dδ1* phospholipases encoding genes at 18 hpi was revealed, whereas the phospholipase *Dγ2* gene was shown to be down-regulated at that time point [68]. Moreover, elevated levels of *A2*, *A2β*, *Dα1* and *Dβ1* phospholipase gene transcripts in *B. cinerea*-infected Arabidopsis plants were detected (Table 1) [69]. Nevertheless, elevated transcript levels for phospholipase *A1γ* and *Dβ1* in wild tomato (*Solanum lycopersicoides*) at 24 h after *B. cinerea* infection were revealed [70]. In lettuce (*Lactuca sativa*), in plants infected with *B. cinerea,* up-regulation of three phospholipase A1 and four phospholipase D (γ1, ζ1, ζ2 and one of unknown isoform) encoding genes were observed at 48 hpi (Table 1) [71]. The up-regulation of phospholipase *A1*, *Dβ1* and *Dα2* genes was detected in pooled samples of chrysanthemum (*Chrysanthemum morifolium*) leaves, collected at five time points between 0 and 72 h after inoculation with *Alternaria tenuissima* [72]. Comparison of transcriptomes of resistant (R) and susceptible (S) *Brassica napus* lines challenged with *S. sclerotiorum* infection revealed significant up-regulation of phospholipase *A2α* and *Dζ2* genes in R lines at 48 h post-inoculation (hpi). No significant increase in expression level of *PLA1* genes was observed in this case [73].

In light of the above results, it has to be considered that trigger-up of jasmonate biosynthesis upon necrotrophic fungi infection is not exclusively regulated by the phospholipase *A1* family genes and that the role of *A2* and especially the *D* family of PLs genes may be underestimated here (Table 1). This conclusion is consistent with the findings of depleted production of JA and resistance level in *PLDβ1* dysfunctional Arabidopsis mutants, suggesting a role of the *Dβ1* phospholipase gene as a positive regulator of JA biosynthesis in response to *B. cinerea* [43].

### 3.2. Lipoxygenase (LOX) Genes

Plant lipoxygenases are often classified according to a positional specificity for the oxygenation of polyunsaturated fatty acids (PUFAs). Thus, plants produce two classes of lipoxygenases 13-LOX and 9-LOX inserting O2 to C-13 or C-9 position of hydrocarbon backbone of linolenic acid, respectively [74]. However, only 13-LOXs participate in JA biosynthesis. From six genes encoding lipoxygenases in *A. thaliana*, four genes encode LOX2, LOX3, LOX4 and LOX6 enzymes that show 13S-lipoxygenase activity, contain chloroplast signaling peptides, and were proven to function in JA biosynthesis in Arabidopsis [1,35,75]. Analysis of RNA sequencing-based transcriptomics revealed that Arabidopsis plants challenged with *B. cinerea* infection displayed the elevated expression of *LOX2* and *LOX4* genes at 18 hpi compared to control plants [67,68]. Quite confusingly, in a previous study, the down-regulation of *LOX2* in Arabidopsis plants starting 20 h after inoculation with *B. cinerea* was observed; however, the *LOX4* gene was shown to be up-regulated within that time [69]. The authors speculated that such differences in regulation of the genes belonging to the same pathway may reflect distinct roles of particular *LOX* genes in the biosynthesis of JA in response to different stimuli. However, *LOX2* down-regulation was also observed in susceptible *Brassica oleracea* inoculated with *A. brassicicola* at a later stage of infection (48 hpi) [76]. In phenotypically resistant *Brassica napus* genotypes, when comparing susceptible plants, the *LOX2* gene was found to be up-regulated at 24 h, whereas *LOX3* and *LOX4* genes were up-regulated at 48 h after inoculation with *S. sclerotiorum* [73,77,78]. Similarly, expression of *LOX2* and *LOX4* genes was induced in lettuce plants inoculated with *B. cinerea* at 48 hpi [71].

Surprisingly, no significant induction of 13S-lipoxygenase genes was observed neither in cucumber (*Cucumis sativa*) [79] nor in *S. lycopersicoides* plants [70] and *S. lycopersicum* fruits [80] infected with *B. cinerea*. However, tomato (*S. lycopersicum*) mutants with a dysfunctional 13S-lipoxygenase D (*TomLOXD*) gene displayed severely compromised resistance to *B. cinerea*. Consistently, the overexpression of *TomLOXD* resulted in elevated JA biosynthesis and enhanced resistance to this pathogen [44]. The above results suggest that in the case of *LOX* genes the regulation of their product abundance may be driven by the mechanism different than transcriptional control.

### 3.3. Allene Oxide Synthase (AOS) and Allene Oxide Cyclase (AOC) Genes

Allene oxide synthase (AOS) catalyzes the synthesis of LOX-produced 9-/13-HPOT (polyunsaturated fatty acids hydroperoxides) to the unstable epoxide, 12,13-EOT (12,13-epoxyoctadecatrienoic acid), which is further cyclized to 12-oxo-phytodienoic acid (OPDA) by allene oxide cyclase (AOC). Similar to LOXs, only 13-AOS functions in JA biosynthesis. Either *13-AOS* and *AOC* genes encode a plastid-transit peptide, indicating that OPDA synthesis is localized in chloroplast [35]. In Arabidopsis, a single copy of *AOS* gene and four genes of *AOC* have been identified [81,82].

The induction of the *AOS* gene in both resistant (R) and susceptible (S) *B. napus* genotypes was revealed at 24 h after inoculation with *S. sclerotiorum*; however, the higher level of its expression was observed in R genotypes at that time point [73]. The up-regulation of *AOS* gene was also observed in Arabidopsis after inoculation with *B. cinerea* (18 hpi) [68], lettuce plants (48 hpi) [71], as well as in green and ripe tomato fruits (1 dpi) [80].

A significant up-regulation of *AOC2* gene was observed in resistant *B. napus* genotypes 48 h after inoculation with *S. sclerotiorum* [78]. Nevertheless, in most recent studies, no significant differences in *AOC2* expression level were found between R and S genotypes for this pathosystem [73]. The latter authors, however, observed the enhanced up-regulation of the *AOC3* gene at 24 hpi and the *AOC4* gene at 48 hpi in *B. napus* R genotypes when compared to S plants. The up-regulation of *AOC2* and *AOC3* genes was observed in Arabidopsis plants 18 h after inoculation with *B. cinerea* [67]. These results are unanimous with previous research on this pathosystem in which the induced expression of *AOC2* and *AOC3* was observed at 8 and 20 hpi, respectively [69]. However, in the latter experiment, the down-regulation of the *AOC4* gene was observed after 20 hpi, similar to the *LOX2* manner of expression yet inconsistent with the other members of this pathway (see Section 3.2). Confusingly, no significant changes were found in any of the *AOC* gene expressions in Arabidopsis plants tested at 18 h after inoculation with *B. cinerea* [68]. Similar to that, no regulation of *AOC* genes was detected in tomato (*S. lycopersicum*) fruits [80] and cucumber (*C. sativa*) plants [79] infected with this pathogen. In the latter case, the operation of a signaling pathway alternative to JA-mediated one may be speculated, as none of the genes involved in jasmonate biosynthesis displayed a regulation in infection-triggered manner.

### 3.4. Oxo-Phytodienoic Acid Reductase (OPR) Genes

The family of oxo-phytodienoic acid (OPDA) reductases (OPRs) comprises at least 3 members in tomato, 6 in Arabidopsis, 6 in pea, 8 in maize and 10 in rice [83]. As described above (Section 2), the silencing of the *OPR3* gene in tomato as well as disruption of *OPR7* and *OPR8* genes in maize resulted in decreased production of JA and diminished resistance to necrotrophic fungi [46,47], supporting the idea that jasmonic acid and not OPDA plays a crucial role in defense to this group of pathogens. The up-regulation of *OPR1* and *OPR3* genes in susceptible and resistant *B. napus* genotypes infected with *S. sclerotiorum* was observed, with no significant differences in expression levels between the two phenotypic groups [73].

Quite unexpectedly, no up-regulation of the *OPR3* gene in Arabidopsis upon infection with *B. cinerea* was revealed. However, 24 h after the combined challenge with *B. cinerea* and herbivore pest *Pieris rapae,* the induction of this gene was observed, suggesting that in that case the mechanical wounding stimulus had a bigger effect on JA biosynthesis than of necrotrophic infection alone [67]. These findings are in accordance with earlier research [69] that also reported no time-course differences in *OPR* genes expression in Arabidopsis plants during *B. cinerea* infection. The explanation for such expression observed in the above-mentioned experiments seems unobtainable at the moment, especially as the up-regulation of the *OPR3* gene was -yet revealed in another transcriptomic study in this pathosystem [68].

## 4. JA Perception and Signal Transduction

The key component of the jasmonate perception apparatus is the F-box COI1 receptor protein, containing N-terminal F-box and Leucine Rich Repeats domains [16], which shows structural resemblance to the auxin receptor protein TIR1 [84]. COI1 is incorporated in the SKP1/CUL1/F-box (SCF) ubiquitin ligase E3 complex, directing its target jasmonate ZIM-domain proteins (JAZ) to degradation via the S26 proteasome pathway [1,19,85]. Our knowledge on the mechanism by which the JA-Ile signal is received and transduced via the SCF-COI1 complex has improved rapidly in recent years. Using the direct ligand-binding assay, the mechanism of JA-Ile mediated interaction between COI1 and JAZ proteins was revealed. It has been proven that JA-Ile promotes the COI1-JAZ interaction in a direct way and that COI1 is an essential component of jasmonate perception machinery [21]. Shortly after, the structural characteristics of COI1 determining its ability to bind the JA-Ile were also revealed and, following that discovery, its ability to interact with JAZ demonstrated that COI1 functions as an immediate receptor of JA-Ile [17]. Resistance of wild type *A. thaliana* to necrotrophic fungi *A. brassicicola* and *B. cinerea* is based on the perception of JA-Ile by the COI1 receptor [50,86,87,88], especially with the knowledge that *coi1-1* and also *jar1* mutants are susceptible to both of these fungi [76,86]. Application of exogenous isoleucine (Ile) enhances wild type Arabidopsis resistance to *B. cinerea,* probably by increasing endogenous JA-Ile. However, in this experiment, *jar1* and *coi1* mutants did not show any reduction in lesions size, proving the substantial role of JA-Ile in the activation of the COI1-JAZ-dependent JA signaling pathway. Exogenous application of Ile to lettuce, strawberry fruits and flowers of red and white roses also moderately enhanced resistance to *B. cinerea*. Moreover, the Arabidopsis mutant *lib* that exhibited a higher content of endogenous Ile was also more resistant to *B. cinerea* than wild type Arabidopsis plants [89].

Regulation of COI1 abundance is essential to exerting its appropriate biological functions *in planta*. In Arabidopsis, COI1 is regulated at the posttranscriptional level and its stability is maintained by the integrity of the SCF-COI1 complex itself. It was revealed that in Arabidopsis mutants impaired in producing SKP1 (ASK1) and CUL1 components of SCF complex, the dissociated COI1 is degraded through the 26S proteasome pathway, suggesting that the COI1 protein is thoroughly regulated by a dynamic balance between SCFCOI1-mediated stabilization and 26S proteasome-mediated degradation [90,91].

Since the research involving the above-mentioned TIR proteins of Arabidopsis, showing a high similarity to the COI1 protein, confirmed that the additional co-factor, namely, inositol hexaphosphate (phytic acid) (InsP6) [92], is involved in hormone binding, similar studies were conducted with regard to the COI1 protein. These studies confirmed that the molecule of inositol pentaphosphate (InsP5) is involved in COI1/JA-Ile/JAZ interaction [84,93]. The function of yet another member of this group, inositol pyrophosphate (InsP8), has been proven to be directly linked with the jasmonate-mediated defense against necrotrophs. Arabidopsis *vih2* mutants with depleted inositol phosphate kinase function (key enzyme in inositol pyrophosphates biosynthesis) were impaired in biosynthesis of InsP8 and showed increased susceptibility to *B. cinerea* and *A. brassicicola* [94].

Inositol phosphate co-factors interact with one arginine residue of JAZ protein and three arginine residues of COI1 protein to form a tetragonal structure inside the complex. Most probably, the presence of the co-receptor promotes higher reactivity of the receptor to the hormone [84]. Additionally, the F-box sequence of the protein itself was revealed as important for binding JA, since a single change of E22A amino acid inside this sequence disables the formation of the SCFCOI1 complex and binding of the hormone molecule [95]. Recently, the coincident detection of jasmonate and inositol phosphates by the SKP1-COI1-JAZ receptor complex was postulated as a mechanism of preventing an uncontrolled accidental activation of immune responses that could severely affect plant growth and development [94].

**Figure 2:**
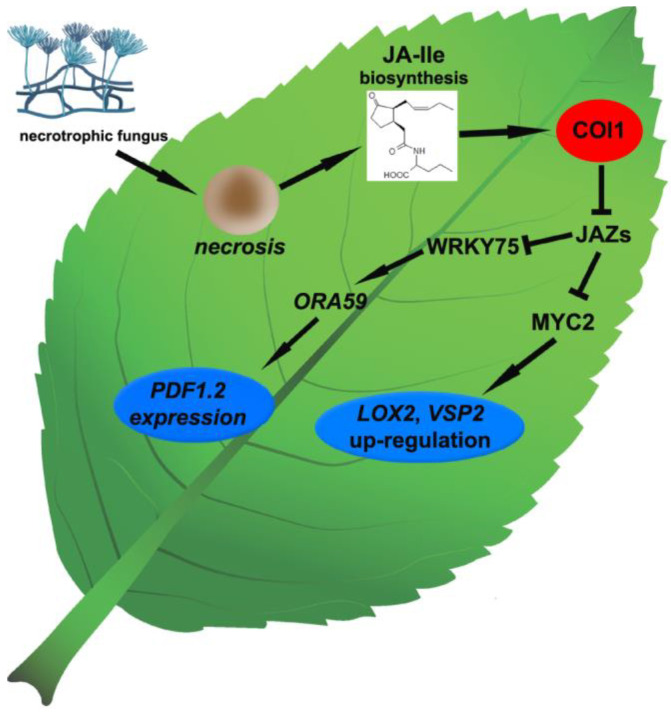
Infection of a plant within foliar tissues with a necrotrophic fungus triggers JA biosynthesis and JA perception via COI1 receptor complex regulated with JAZs and subsequent JA-dependent signal transduction through transcription factors such as WRKY75 and/or MYC2 and downstream expression of defense-related genes such as *PDF1.2*, *VSP2* and *LOX2*. JA-responsive gene *ORA59* was detected exclusively as being activated downstream WRKY75 [12,112,120]. Detailed description in the text.

Aside from the COI1 protein, the family of JAZ repressor proteins is also crucial for the expression of JA-dependent genes during defense response (Figure 2) [19]. In *A. thaliana*, the family of JAZ proteins has 12 members [85] possessing two characteristic regions: a C-terminal Jas domain and a centrally located ZIM domain, which are responsible for the formation of protein homo- and heterodimers [96]. The role of JAZ in regulation of JA signaling in response to necrotrophic fungi has been confirmed in Arabidopsis during its interactions with *B. cinerea* or *Fusarium oxysporum* [87,97]. *Botrytis cinerea* infection caused larger necrotic lesions in Arabidopsis *jaz-6* mutant than in wild type plants [87]. However, the moderately resistant wild type Arabidopsis infected with *F. oxysporum* showed elevated expression of *JAZ6*, *JAZ7*, *JAZ9* and *JAZ12* genes, although constitutive *JAZ* expression in Arabidopsis mutant enhanced susceptibility to the pathogen [97]. Moreover, susceptible orchid plants (*Dendrobium catenatum*) challenged with a Southern Blight fungus *Sclerotium delphinii* showed higher expression of *JAZ* genes (*DcJAZ1*, *DcJAZ2*, *DcJAZ4* and *DcJAZ5*) compared to the control plants [98].

Degradation of JAZs releases their downstream transcription factors, including MYC2/3/4 [99,100,101], MYB21/24 [102] and WD-repeat/bHLH/MYB [103], to mediate jasmonate responses. The Jas motif is responsible for the interaction with the COI1 protein as well as with the MYC2 transcription factor [20]. Moreover, studies on Arabidopsis revealed that JAZ proteins need to bind to their co-repressor TOPLESS (TPL) either directly or indirectly by NINJA (Novel Interactor of JA) adaptor protein, a repressor of transcription of JA-responsive genes [104,105,106]. The new member of the JAZ family (JAZ13) was described as interacting with transcription factor MYC2 and co-repressor TOPLESS; however, due to the unusual structure of the ZIM domain, it was unable to interact with NINJA [107]. It has been reported that plant immunity was regulated via co-repression by members of the TOPLESS family (*TLP1*, *TPR1*) plant resistance proteins or pathogen effectors promoting either plant resistance or susceptibility, respectively. However, the studies were concerned mostly about plant responses to bacteria *Pseudomonas syringae* (strain Pst DC3000) and oomycete *Hyaloperonospora arabidopsidis* [106,108,109].

The MYC2 transcription factor, belonging to the bHLH (basic Helix-Loop-Helix) family, binds G-box and G-box-like sequences within the promoters of jasmonic acid-regulated genes. MYC2 can induce response to injury, biosynthesis of JA and adaptation to oxidative stress or inhibit JA-dependent tryptophan metabolism and response to pathogenic fungi [110]. It was observed that this dual nature of the MYC2 factor, capable of both induction and inhibition of JA-dependent gene expression, is probably dependent on the presence of other hormones. MYC2 up-regulates genes whose expression requires the presence of JA itself, e.g., *VSP2* and *LOX2* genes (Figure 2). The MYC2-dependent inhibition of gene expression takes place whenever the synergistic effect of two or more hormones is required for the induction of respective genes, e.g., in the case of *PDF1.2* marker gene, which requires the co-presence of JA and ethylene [111]. Otherwise, MYC2 is considered as a marker transcription factor for the JA-dependent signaling pathway and its expression has been observed in many necrotrophic pathosystems (Figure 2) [112]. In host plant response to necrotrophic fungi, *MYC2* is often over-expressed, especially at early stages of infection. The up-regulation of *MYC2* appears even in susceptible plants, mostly before lesions spreading. Thus, *MYC2* was over-expressed together with elevated JA content in poplar (*P. davidiana* × *P. bollena*) infected with *Alternaria alternata* at 2 dpi, but with the disease progression *MYC2* level decreased as well as JA content [113]. A similar pattern of *MYC2* expression, decreasing with lesion spread, was noticed in susceptible apple cultivar in response to *A. alternata* [114]. Consistently, cucumber cultivar susceptible to *B. cinerea* showed only a slight increase in *MYC2* expression, whereas its expression in resistant cultivar significantly increased in a time-dependent manner [115].

Aside from MYC2, other transcription factors from the bHLH family, MYC3 and MYC4, were also identified [100]. MYC3 and MYC4 show high amino acid sequence analogy to MYC2 and are present in the nucleus [99,101]. Both MYC act together and are required for the full hormonal response to pathogenic bacteria and herbivorous insects [100]. Moreover, MYC2, MYC3 and MYC4 mediate JA-mediated defenses in Arabidopsis against *B. cinerea* [116]. More recently, another group of bHLH transcription factors JAM (Jasmonate Associated MYC2-like) was described, negatively regulating other transcription factors involved in the expression of JA-responsive metabolic genes [117,118]. All MYC transcription factors are capable of interacting with JAZ proteins, but the strength of this interaction can be variable or even lacking depending on the type of proteins involved. MYC2 was revealed to be able to interact with all twelve JAZ proteins, while MYC3 can interact with only eight of them [99,100]. Moreover, other MYC factors, still not described in detail, i.e., MYC5, MYC13 and MYC17, can interact with JAZ proteins through the N-terminal end that is characteristic for this family [101]. However, the potential role of these MYCs in plant response to necrotrophic fungi has not been explored yet.

Although many JA-responsive transcription factors other than members of the bHLH family function in regulating various aspects of plant metabolism and physiological processes such as WRKY or MYB [12,119], only a few of them, however, were recognized as being involved directly in plant defense response to necrotrophic fungi. One such TF, WRKY75, was recognized to be a positive regulator of JA-mediated defense response that interacts with JAZ8, which represses its transcriptional function. Upon infection of Arabidopsis with *B. cinerea*, production of endogenous JA induced degradation of JAZ8 and released WRKY75. Afterward, WRKY75 activated expression of JA-responsive *ORA59* gene and downstream defense genes such as *PDF1.2* (Figure 2) [120]. In the case of wild type cotton (*Gossypium hirsutum*) infection with *V. dahlia*, the GhMYB4 transcription factor positively regulates the resistance to the fungus by blocking lignin deposition through direct suppression of the expression of genes involved in lignin synthesis. Consequently, reduction of lignin may result in alteration of cell wall integrity and subsequently more oligogalacturonides is released, which may activate JA biosynthesis and defense responses in cotton. However, this hypothesis has to be confirmed yet [121].

## 5. JA-Mediated Response to Necrotrophic Infection Regulated by Circadian Clock and Photoperiod

The circadian clock, an endogenous time-keeping mechanism, adjusts biological processes of a plant in response to environmental signals, so that they are turned on at optimum times throughout the day [122,123]. Plant defenses are also rhythmically regulated to be expressed with full strength at the time of maximal susceptibility to infection or to synchronize with the time of the day when a pathogen is most abundant [124]. Arabidopsis plants show differential susceptibility to *B. cinerea* depending on the time of inoculation during the day [125]. It is speculated that plants can anticipate the timing of pathogen infection by time-specific defense pathway activation and thus maximize the response against a particular pathogen [126]. Consequently, the susceptibility of Arabidopsis to *B. cinerea* decreases after inoculation at early daytime (dawn) compared with night. Moreover, the state of decreased susceptibility persists under permanent light conditions and is disrupted in mutants impaired in circadian clock (CC) function. Moreover, the enhanced susceptibility to this pathogen has been lost in the *jaz6* mutant, suggesting the key role of JA signal transduction via JAZ6 in rhythm-dependent susceptibility of Arabidopsis to *B. cinerea* [125]. As yet, the only evidence for the direct molecular interaction between CC and JA-mediated defense components comes from the plant response to bacteria *P. syringae* pv. *tomato*. As it was revealed, the circadian clock component TIME FOR COFFEE (TIC) rhythmically regulates the JA signaling pathway in Arabidopsis by inhibiting MYC2 protein accumulation and controlling transcriptional repression of COI1 in an evening-phase-specific manner [126]. In case of temporal variation in susceptibility to necrotrophic fungi, the operation of more complex functional CC network has been suggested, since among the transcription factors that responded more rapidly to infection at subjective dawn than subjective night, the target genes of core clock regulators were shown to be notably abundant [125]. Moreover, duration of the light period seems to influence not only regulation of plant response to biotic stress factors but also the development of an attacking pathogen [127,128]. Mustard plants (*B. juncea*) grown under different regimes of light periods showed variation not only in leaf size but also in necrosis formation in response to *A. brassicicola*. The light period over 16 h restricted leaf development and necrosis spreading [129]. However, how this phenomenon may be connected to a plant JA-dependent resistant response to *A. brassicicola* must be further explored [129]. Consistently, long day photoperiod enhanced Arabidopsis resistance to *B. cinerea* activating JA-dependent defense responses, e.g., expression of *MYC2* gene [130]. Nevertheless, the JA-dependent influence of circadian clock and photoperiod on defense response to necrotrophic fungi requires further extensive investigations.

## 6. JA Signaling Regulating Trade-Off between Defense and Growth Strategies

Biotic stresses, such as competition with adjacent plants or pathogens/insects attack, are the main agents limiting the fitness of plant. Thus, addressing the resources for growth can limit their availability for defense and vice versa their allocation to defense can reduce growth and competitiveness against neighboring plants. Therefore, plants must maintain a dynamic balance in their responses to these sources of stress, and when challenged with pathogen attack, they must allocate their metabolic resources to defense at the expense of growth [131]. This allocation requires a switch between pathways mediated by gibberellic acid (growth) and jasmonic acid (defense). Gibberellins (GAs) are plant hormones regulating plant growth and developmental transitions in response to endogenous and environmental stimuli. Many studies revealed that gibberellins (GA) are involved in JA-dependent signaling pathways during plant response to stress. DELLA proteins are known as plant transcriptional repressors whose degradation is promoted by GA [132,133]. As it was shown in Arabidopsis, JA rapidly induces the expression of the *REPRESSOR OF GA-LIKE3* member of DELLA family (*RGL3*), and this induction is triggered in a CORONATINE INSENSITIVE1 (COI1)- and JASMONATE INSENSITIVE1 (JIN1/MYC2)-dependent manner [134]. In the absence of GA, DELLA proteins interact directly with JAZ1, averting its interaction with MYC2 transcription factor and thus promoting MYC2 activity towards regulation of JA-responsive genes [134,135]. Conversely, the presence of GA results in degradation of DELLA and release of JAZ; the latter molecule can bind to the MYC2 factor, inhibiting the expression of genes associated with the JA pathway [135]. The transcription factor proteins microarray analyses revealed that DELLA proteins RGL1, RGA1 (REPRESSOR OF GA) and GAI1 (GA-INSENSITIVE) also interact directly with MYC2. This finding provides an alternative mechanistic model of cooperation between JA and GA signaling pathways in which interaction with DELLAs protects MYC2 from inhibition by JAZ [136].

Expectedly, DELLAs were also shown to be involved in the molecular mechanism underlying the inhibition of growth during JA-mediated defenses. In both monocot rice and dicot Arabidopsis, combinative analysis of *coi1*, *della* and *pif* (phytochrome interacting factor—growth promoting transcription factor) mutants revealed that the molecular signal for the transition from growth to defense strategy is transmitted through the COI1–JAZ–DELLA–PIF signaling module [137]. In complement to this finding, the mapping of the TF interactome revealed that the JA/ET-dependent transcription factor EIN3 also interacts with PIF4, as well as with DELLA (RGA1) protein, providing a new insight into the mechanism of cooperative JA/ET/GA-mediated signaling [136]. Accordingly, DELLA mutant (*rgl3-5*) displayed increased susceptibility to *B. cinerea*, clearly referring to the involvement of GA signaling in JA-mediated defense [134]. In consistency with this, *RGL3* (DELLA) gene expression was found significantly induced in Arabidopsis plants infected with *B. cinerea*, whereas the expression of JA-responsive marker genes including the pathogen-responsive *PLANT DEFENSIN1.2* (*PDF1.2*) and *ETHYLENE RESPONSE FACTOR1* (*ERF1*) genes in *rgl3-5* infected with this pathogen was broadly inhibited [134,138].

However, simultaneous exposure to pathogen attack and limited light conditions force the plant to balance the costs of controlling each stressor and to choose the most effective developmental strategy. The decrease of red:far red (R:FR) light ratio occurring in dense canopies favors growth to outcompete adjoining plants but has a restraining effect on JA-mediated defenses [139]. Although a molecular link between the light signal and JA-mediated defenses against necrotrophs remains unascertained, the report provided some insight into this interrelation. From the analysis of COI1-defective Arabidopsis mutants (*coi1*), it was revealed that the low R:FR depressing effect on defense against *B. cinerea* requires the operation of the SCFCOI-JAZ JA receptor complex [140]. Furthermore, JA-responsive MYC2, MYC3 and MYC4 transcription factors were shown to be essential for JA-mediated defenses against *B. cinerea*, as well as for the shade-triggered increased susceptibility, indicating that shade conditions may negatively affect the defense by mediating the inactivation of MYC transcription factors. In fact, phytochrome B inactivation by shade (light spectrum enriched with far red) not only destabilizes these three proteins but also reduces their stabilization by JA. As opposed to MYCs, shade conditions were proven to stabilize JAZ repressors and reduce their degradation by JA [116,141]. Moreover, JA signaling in plants exposed to low R:FR ratios is additionally repressed by degradation of DELLA proteins and thus reduces their availability for interaction with JAZs [141]. In addition, the integrated metabolomics and transcriptomics studies on Arabidopsis revealed that suppression of Arabidopsis defense against *B. cinerea* in shade conditions is mediated by reduced levels of tryptophan-derived glucosinolates [142]. However, the possible interrelation between glucosinolate and the JA pathway remains obscure in this context. As expected, the contrary regulation of MYCs and JAZs proteins stability resulting from the inactivation of phytochrome B, down-regulates the expression of defense markers responsive to *B. cinerea*, including the genes encoding ERF1 and PDF1.2 [139,140]. Moreover, low R:FR ratios suppresses not only JA-dependent but also SA-dependent genome-wide transcription profiles, suggesting that entering the competition-for-light mode brings about the attenuation of defenses in general [139]. Consistently, the exposure to low R:FR ratios, as well as the phyB mutation, significantly increase plant susceptibility to both bacteria *P. syringae* (SA-dependent defense) and necrotrophic fungus *B. cinerea* [139,140].

## 7. JA and ET Signaling Pathways Cooperation in Immune Response to Necrotrophic Fungi

JA- and ethylene (ET)-mediated signaling pathways play an important role in defense against necrotrophic fungi, and these two hormones mediate the immune response in both independent and synergistic manners [143,144]. On the molecular level, the synergism between JA and ET pathways consists in the physical interaction of JA-dependent JAZ repressor proteins with ETHYLENE INSENSITIVE3 (EIN3) and EIN3-LIKE1 (EIL1) transcription factors, which are the key regulators of ET-mediated responses. The presence of JA-Ile promotes the degradation of JAZ and thus leads to the release of EIN3 and EIL1, which in turn require ET for their stabilization [145]. Noteworthy, ET-dependent EIN3/EIL1 and JA-dependent MYC (MYC2, MYC3, MYC4) transcription factors are reciprocal repressors, and their physical interaction regulates antagonism between responses mediated exclusively by JA (e.g., against herbivore attack) and JA/ET cooperative responses against necrotrophic pathogens [146]. Releasing from JAZ repression and ET-stabilized EIN3/EIL1 positively regulates their downstream transcription factors ERF and ORA59, which interact subsequently with promoters of necrotrophic pathogens-responsive genes, such as *PDF1.2* [147]. Moreover, the transcriptomic analyses revealed that the plant defensin (*PDF*) family genes are among the most distinctly up-regulated in Arabidopsis plants constitutively expressing *ERF5* and *ERF6* genes [148]. The overexpression of yet another member of the ERF family (*ERF96*) in Arabidopsis plants was shown to enhance the expression of JA/ET-dependent defense genes *PDF1.2a*, *PR-3*, *PR-4*, and this enhancement was proved to be mediated via direct binding of ERF96 to GCC elements of these genes’ promoters [149]. Quite expectedly, Arabidopsis plants overexpressing any of the *ERF* genes mentioned above also demonstrated elevated resistance to *B. cinerea* [148,149]. Moreover, Arabidopsis phytoalexin, camalexin, is required for resistance to both *B. cinerea* and *A. brassicicola* in wild type plants [150,151]. Recently, it was reported that JA and ET induce synergistically via ERF1 pathogen-responsive camalexin biosynthesis [152].

It has to be emphasized that the JA-dependent signaling pathway is only a part of a plant’s active response to stress. Therefore, synergy or antagonism during interaction of JA with other phytohormones such as brassinosteroids, auxins, abscisic acid (ABA) and salicylic acid (SA) have been extensively studied, but such research is rather rarely performed and described exclusively in the context of JA-dependent plant response to necrotrophic fungi [153,154,155].

## 8. Conclusions

Negative impact of climatic changes and a growing human population requires harnessing new efficient technologies in agriculture to increase yield of crops and decrease to minimum the loss of yield and incomes due to the disadvantageous influence, among other factors, of pathogenic fungi [156]. One of the new approaches to create modern agricultural technologies, which fit into ecological trends leading mostly in Europe and North America, is the use of natural plant defense mechanisms against pathogens. Skilled use and/or manipulation of JA biosynthesis and JA-dependent signaling pathways can be a good basis for development of novel ‘green’ compounds that not only stimulate growth of plants but also increase the defense capacity of the whole plant with a long-lasting effect against attacks of various necrotrophic pathogens.

In recent years, many research groups all over the world have worked on JA biosynthesis and signaling in various crop species. However, further investigations should also focus exclusively on the JA-dependent signal transduction pathway and JA-responsive genes activation in plants resistant and susceptible to necrotrophic fungi under not only laboratory conditions but also in the field.

## Figures and Tables

**Table 1:** Phospholipase (*PL*) genes encoding different isoforms of PLA and PLD active in various pathosystems.

Phospholipase Gene	Pathosystem	References
*A1, A2,* A2α *A2β,* *Dα1, Dβ1, Dγ1, Dδ1*	*A. thaliana*-*B. cinerea*	[67,68,69]
*A1γ, Dβ1*	*S. lycopersicoides*-*B. cinerea*	[70]
*A1*, *Dγ1*, *Dζ1*, *Dζ2*	*L. sativa-B. cinerea*	[71]
*A1, Dβ1*, *Dα2*	*C. morifolium-A. tenuissima*	[72]
*A2α, Dζ2*	*B. napus-S. sclerotiorum*	[73]

## Data Availability

Not applicable.

## Abbreviations

ABA	abscisic acid
ACX	acyl-CoA oxidase
AOC	allene oxide cyclase
AOS	allene oxide synthase
bHLH	basic Helix-Loop-Helix motif
CC	circadian clock
COI1	CORONATINE-INSENSITIVE1
CWDE	cell wall degrading enzyme
EIL1	EIN3-LIKE1
EIN3	ETHYLENE INSENSITIVE3
ERF1	ETHYLENE RESPONSE FACTOR1
ET	ethylene
12,13-EOT	12,13(S)-epoxy-9(Z),11,15(Z)-octadecatrienoic acid
Fhb1	*Fusarium* head blight
GAs	gibberellins
GAI1	GA-INSENSITIVE1
13-HPOT	(13S)-hydroperoxy-octadecatrienoic acid
InsP5	inositol pentaphosphate
InsP6	inositol hexaphosphate
InsP8	inositol pyrophosphate
(+)-7-iso-JA	(+)-7-iso-jasmonic acid;
(+)-7-iso-JA-Ile	(+)-7-iso-jasmonoyl-L-isoleucine;
JA	jasmonic acid, jasmonate(s)
JA-Glc	jasmonic acid glucoside
JA-Ile	JA-isoleucine conjugate
JA-Phe	jasmonoyl-phenylalanine
JAR1	Jasmonate-Resistant synthase
JAZ	Jasmonate-ZIM (Zinc-finger Inflorescence Meristem)
JIN1	JASMONATE INSENSITIVE1
JMT	JA carboxyl Methyl Transferase
KAT	l-3-ketoacyl-CoA thiolase
α-LeA	α-linolenic acid
Leu	leucine
LOX	lipoxygenase
MeJA	methyl ester of JA
MYB	transcription factor
MYC	transcription factor
NINJA	Novel Interactor of JA
12-OH-JA	12-hydroxyjasmonic acid (tuberonic acid)
OPC-8	3-oxo-2-(2′-[Z]-pentenyl)cyclopentane-1-octanoic acid
OPDA	cis-(+)-12-oxo-phytodienoic acid
OPR	oxo-phytodienoic acid reductase
PDF	plant defensin
Phe	phenylalanine
Pif	phytochrome interacting factor
PL	phospholipase
PLA1	phospholipase A1
PR	pathogenesis-related gene/protein
RGL	REPRESSOR OF GA-LIKE3. member of DELLA family
ROS	reactive oxygen species
SA	salicylic acid
SCF	SKP1/CUL1/F-box
SSITL	*Sclerotinia sclerotiorum* integrin-like
TIC	TIME FOR COFFEE
THI	thionin
TIR1	auxin receptor
TPL	TOPLESS
Val	valine
VSP	vegetative storage protein
WRKY	transcription factor
